# Bibliography

**Published:** 1904-09-15

**Authors:** 


### BIBLIOGRAPHY.

ESSIG'S DENTAL METALLURGY.—A new and fifth edition of this valuable work has just come from the press of Lea Bros. & Co. The work has been carefully revised and made to conform to modern knowledge by Dr. Augustus Koenig, of the University of Pennsylvania. Considerable new material has been added, likewise many new illustrations, all for the purpose of bringing the book up-to-date and making it more acceptable as a laboratory and class-room guide. The work has, for a long time, been recognized as the best book extant on its subject. It has surely not suffered at the hands of its present editor.

DUNHAM'S NORMAL HISTOLOGY.—This, the third edition of this popular work, has just been issued by Lea Bros. & Co. It is a beautifully printed volume of over three hundred pages, well illustrated. It is not a treatise, but designed to present the subject in a systematic and brief manner, so that the student may secure a clear conception of the subject in the time generally allowed for the study of this important subject. The commendable characteristics of this book are its orderly presentation of the subject and the clear and intelligible statement of its matter.

Diagramatic figures assist wonderfully in comprehending and fixing on the memory the text. Very little attention is given to the teeth, but, as a basis of general histology, is it a good work.

